# Isotopic niche differs between seal and fish‐eating killer whales (*Orcinus orca*) in northern Norway

**DOI:** 10.1002/ece3.6182

**Published:** 2020-04-08

**Authors:** Eve Jourdain, Clare Andvik, Richard Karoliussen, Anders Ruus, Dag Vongraven, Katrine Borgå

**Affiliations:** ^1^ Norwegian Orca Survey Andenes Norway; ^2^ Department of Biosciences University of Oslo Oslo Norway; ^3^ Norwegian Institute for Water Research Oslo Norway; ^4^ Norwegian Polar Institute Tromsø Norway

**Keywords:** ecological niche, ecological variation, prey specialization, stable isotopes, trophic level

## Abstract

Ecological diversity has been reported for killer whales (*Orcinus orca*) throughout the North Atlantic but patterns of prey specialization have remained poorly understood. We quantify interindividual dietary variations in killer whales (*n* = 38) sampled throughout the year in 2017–2018 in northern Norway using stable isotopic nitrogen (δ^15^N: ^15^N/^14^N) and carbon (δ^13^C: ^13^C/^12^C) ratios. A Gaussian mixture model assigned sampled individuals to three differentiated clusters, characterized by disparate nonoverlapping isotopic niches, that were consistent with predatory field observations: seal‐eaters, herring‐eaters, and lumpfish‐eaters. Seal‐eaters showed higher δ^15^N values (mean ± *SD*: 12.6 ± 0.3‰, range = 12.3–13.2‰, *n* = 10) compared to herring‐eaters (mean ± *SD*: 11.7 ± 0.2‰, range = 11.4–11.9‰, *n* = 19) and lumpfish‐eaters (mean ± *SD*: 11.6 ± 0.2‰, range = 11.3–11.9, *n* = 9). Elevated δ^15^N values for seal‐eaters, regardless of sampling season, confirmed feeding at high trophic levels throughout the year. However, a wide isotopic niche and low measured δ^15^N values in the seal‐eaters, compared to that of whales that would eat solely seals (δ_N‐measured_ = 12.6 vs. δ_N‐expected_ = 15.5), indicated a diverse diet that includes both fish and mammal prey. A narrow niche for killer whales sampled at herring and lumpfish seasonal grounds supported seasonal prey specialization reflective of local peaks in prey abundance for the two fish‐eating groups. Our results, thus, show differences in prey specialization within this killer whale population in Norway and that the episodic observations of killer whales feeding on prey other than fish are a consistent behavior, as reflected in different isotopic niches between seal and fish‐eating individuals.

## Graphical abstract

Interindividual dietary variations were quantified in killer whales (*n* = 38) sampled throughout the year in northern Norway using stable isotopic nitrogen (δ15N: 15N/14N) and carbon ratios (δ13C: 13C/12C). Isotopic niche width and trophic level indicated dietary structuring and differences in prey specialization within this killer whale population. While some individuals appear as conspicuous specialized fish‐eaters, others seem to adopt a diverse diet that includes both fish and mammal prey.

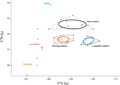

## 1 INTRODUCTION

The killer whale (*Orcinus orca*) is an apex predator found in all the world's oceans (Forney & Wade, 2006). Although a generalist species, studies have revealed discrete prey specializations of sympatric populations in some regions (e.g., Ford et al., 1998; Saulitis, Matkin, Barrett‐Lennard, Heise, & Ellis, 2000) with foraging behaviors apparently culturally transmitted through generations within matrilineal social units (Ford & Ellis, 2014; Riesch, Barrett‐Lennard, Ellis, Ford, & Deecke, 2012). Through cultural divergence and social isolation of specialized groups, prey specialization may influence population structure (Hoelzel et al., 2007; Riesch et al., 2012), and eventually facilitate ecotype formation (Foote et al., 2016). Due to different patterns of resource use, killer whale populations may be differentially impacted by human activities. In the coastal waters of the northeastern Pacific, so‐called resident killer whales rely on salmonids as a main food source while transient killer whales appear to feed exclusively on marine mammal prey (e.g., Ford & Ellis, 2014; Ford et al., 1998). The resident and transient killer whale communities constitute distinct populations and ecotypes (Morin et al., 2010). Divergent demographic trends for these populations since the mid‐1990s have resulted in disparate conservation status and management strategies (COSEWIC, 2008). This is an example of why understanding interindividual diet variations may be important in the management of this species.

In the North Atlantic, at least two types of killer whales differing in morphology, tooth wear and nitrogen isotopic values have been suggested (Foote, Newton, Piertney, Willerslev, & Gilbert, 2009). A far‐ranging generalist so‐called Type 1 includes herring‐feeding killer whales off Norway and Iceland, but with interindividual variation in the dietary proportions of contributing prey items, including high trophic level prey (Foote et al., 2009). This suggestion was supported by a variation in intrapopulation ecological niche in Iceland (Samarra et al., 2018; Samarra, Vighi, Aguilar, & Vikingsson, 2017), and field observations of a subset of individuals switching between pinniped and fish prey from both Norway (Vongraven & Bisther, 2014) and Iceland (Foote, Similä, Vikingsson, & Stevick, 2010). In contrast, larger Type 2 killer whales appear to specialize on cetacean prey (Foote et al., 2009). However, current classification in two types may be oversimplistic considering the large diversity of ecological/dietary patterns across the North Atlantic (see Jourdain, Ugarte, et al., 2019 for a review). Foraging strategies of North Atlantic killer whales at the group and individual levels remain poorly understood.

Killer whales in Norway have historically been thought to specialize on herring (*Clupea harengus*) and to mainly associate with the most abundant Norwegian Spring Spawning (NSS) stock (Similä, Holst, & Christensen, 1996), as supported by concurrent observations. Herring made up almost the entire stomach contents of killer whales caught prior to 1980 (Christensen, 1982, 1982), and killer whales typically occur in large seasonal aggregations at herring wintering grounds (Bisther & Vongraven, 1995; Similä et al., 1996), where they display remarkably specialized feeding behaviors (Domenici, Batty, Similä, & Ogam, 2000; Similä & Ugarte, 1993). However, as killer whale studies have primarily been conducted at herring wintering grounds until recently, other prey utilized in other areas were unlikely to be identified. Research efforts extended to other seasons and regions in Norwegian waters have documented new prey species, for example, the Atlantic mackerel (*Scomber scombrus*, Nøttestad et al., 2014), the Atlantic salmon (*Salmo salar*, Vester & Hammerschmidt, 2013), the harbor porpoise (*Phocoena phocoena*, Cosentino, 2015), and the gray (*Halichoerus grypus*) and harbor (*Phoca vitulina*) seals (Jourdain, Vongraven, Bisther, & Karoliussen, 2017; Vongraven & Bisther, 2014). However, the lack of identification data and/or only brief periods of data collection for these studies precluded any assessment of dietary differences and specializations among killer whale individuals/groups.

Efforts began in 2013 to investigate feeding habits at the individual level, combining predation records of photo‐identified individuals, behavioral observations, and tissue samples collected throughout multiple years (Jourdain, Karoliussen, Vos, Zakharov, & Tougard, 2019; Jourdain et al., 2017, this study). Results revealed that some social groups specialize to some extent on pinnipeds (Jourdain et al., 2017) and that some herring‐eating individuals seasonally switch to feeding on locally abundant lumpfish (*Cyclopterus lumpus*) in spring (Jourdain, Karoliussen, et al., 2019). Social interactions among sympatric killer whales adopting distinct foraging behaviors have not been investigated to date. Field observations provide only a snapshot of observable feeding bouts; combining observational records with time‐integrated dietary markers would assist in assessing interindividual variations in diet, as well as persistency of prey specialization over longer periods of time.

Because the isotopic composition of a predator's tissue reflects that of its prey resources in a predictable/quantifiable manner (DeNiro & Epstein, 1978), stable isotopes have been commonly used in dietary studies (see Newsome, Clementz, & Koch, 2010 for a review). Due to a greater retention of the heavier ^15^N isotope than the lighter ^14^N isotope in the production of nitrogenous waste, the nitrogen ratio of ^15^N to ^14^N (δ^15^N) shows a stepwise enrichment from food source to consumer and is therefore indicative of relative trophic position (DeNiro & Epstein, 1978; Hobson & Clark, 1992). The carbon ratio of ^13^C to ^12^C (δ^13^C), primarily reflects variable carbon origins of inshore/benthic versus pelagic/offshore sources, therefore allowing for discrimination between feeding locations in marine systems (Hobson, Piatt, & Pitocchelli, 1994). Typically, inshore/benthic ecosystems are characterized by higher δ^13^C values than pelagic/offshore systems. Isotopic variance within a population, characterized by variance in the δ^13^C and δ^15^N values of its individuals, can be used as a measure of diet variation, referred to as niche width (Bearhop, Adams, Waldron, Fuller, & Macleod, 2004). Typically, consumers that feed on a wide range of food sources will display larger variations in isotopic signatures and thus a wider niche than prey specialists that feed on a narrow range of prey. Similarly, consumers that feed in multiple locations will show greater variations in δ^13^C, that is, a wider niche (Bearhop et al., 2004).

In the study of killer whales, high variability in δ^15^N and δ^13^C values has proved effective in assessing inter and intrapopulation dietary variations and preferences (Durban, Fearnbach, Burrows, Ylitalo, & Pitman, 2017; Herman et al., 2005; Krahn et al., 2007; Reisinger et al., 2016; Samarra et al., 2017; Tixier et al., 2019). Isotopic profiles confirmed dietary segregation between sympatric resource specialists in the northeastern Pacific (Herman et al., 2005) and around the Antarctic Peninsula (Durban et al., 2017) but also revealed generalist killer whale populations adopting a mixed diet including both fish and mammal prey (Reisinger et al., 2016; Tixier et al., 2019). In these studies, a priori knowledge of sampled individuals/populations through previous field observations has been highly beneficial for meaningful interpretation of dietary patterns (Newsome et al., 2010).

In this study, we use δ^15^N and δ^13^C values from killer whale skin samples collected throughout the year in northern Norway. The aims were to (a) measure interindividual variations in dietary habits by comparing isotopic profiles, that is, trophic level and niche width, of fish and seal‐eating killer whales; (b) estimate the contribution of pinniped prey to the diet of seal‐eating killer whales. Results are discussed in light of predation records available for the sampled whales to assess consistency in individuals’ dietary habits and further evaluate the degree of prey specialization for these whales.

## 2 MATERIALS AND METHODS

### 2.1 Sample collection

**FIGURE 1:**
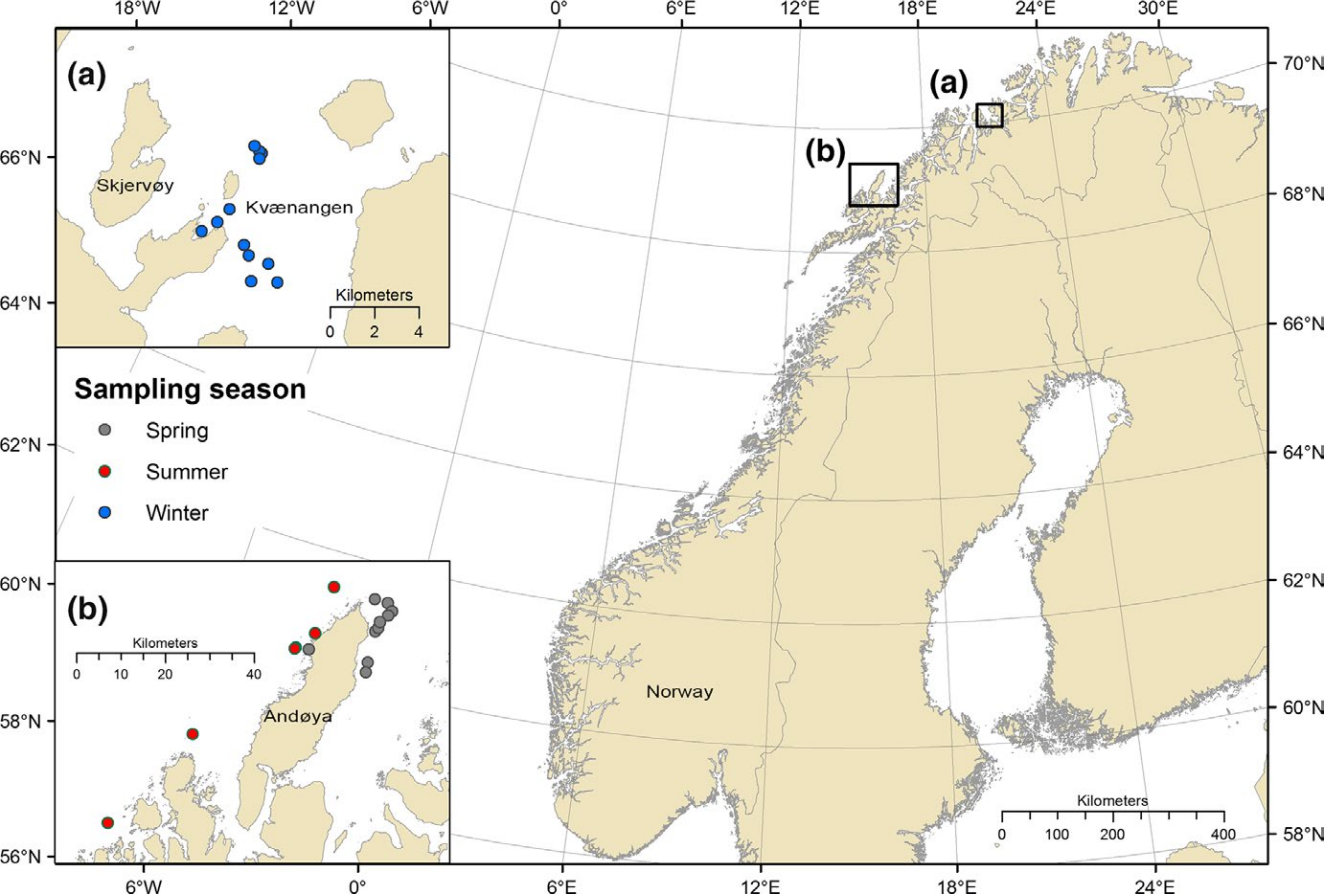
Locations of the 38 killer whale biopsy samples collected in northern Norway in 2017–2018. Region (a) corresponds to Kvænangen, Skjervøy, the herring wintering grounds where killer whales were sampled in November 2017. Region (b) corresponds to Andøya, where whales were sampled at the lumpfish spring spawning grounds and throughout the summer in 2018. Blue dots (*n* = 22) correspond to individual whales sampled during November and assigned to sampling season group *Winter*. Gray dots (*n* = 10) correspond to whales sampled in April–May and assigned to sampling season group *Spring*. Red dots (*n* = 6) correspond to whales sampled in June–August and assigned to sampling season group *Summer*. Herring muscle (*n* = 4) was obtained in region (a). Lumpfish muscle (*n* = 5) and seal muscle (*n* = 1) were obtained from region (b)

Killer whale biopsy samples were collected in August and November 2017 and from April through July 2018 in northern Norway. In November, samples were collected in Kvænangen, Skjervøy (Figure 1a). This fjord was part of the herring wintering grounds (ICES, 2018), and killer whales were observed foraging on herring at time of sampling. The rest of the year, samples were collected off Andøya (Figure 1b), where killer whales were encountered throughout the year and are known to seasonally feed on various prey types including herring (Jourdain & Vongraven, 2017), pinnipeds (Jourdain et al., 2017), and lumpfish (Jourdain, Karoliussen, et al., 2019).

Killer whale biopsies were sampled using an ARTS darting system (Restech) and 25 × 9 mm or 40 × 9 mm stainless steel tips in 2017, and with an injection gun (Pneu‐Dart Inc) and 25 × 7 mm tips in 2018. The biopsy tips were sterilized with boiling water and 95% ethanol and placed in clean plastic bags before use. Killer whales were sampled when travelling or feeding. The region directly posterior to the dorsal fin of adult and subadult killer whales was the target area for sampling. For each sampled individual, identification photographs were taken. Biopsy darts containing skin and blubber were retrieved, stored in a clean plastic bag and placed in a cooling box while at sea. Onshore, skin and blubber layers were sliced apart and stored separately at −20°C until analysis. Isotopic values measured in killer whale skin are expected to represent the individuals’ diet in the four to six weeks prior to sampling. This is based on controlled diet experiments that estimated half‐time turnover rates for bottlenose dolphin (*Tursiops trucuntus*) skin to be 24 ± 8 d for carbon and 48 ± 19 d for nitrogen (Giménez, Ramírez, Almunia, Forero, & Stephanis, 2016).

Estimating the diet of consumers requires isotopic prey values. Because isotopic values may vary greatly both in space and time, it is important that both prey and killer whales are sampled within matching geographic areas and time periods (Phillips et al., 2014). Herring muscle (*n* = 4) was collected in November 2017 in Kvænangen, Skjervøy, lumpfish muscle (*n* = 5) was collected in March–April 2017 in Andfjord while conducting focal studies of feeding killer whales (Jourdain, Karoliussen, et al., 2019). Muscle from a dead stranded harbor seal was sampled in November 2017 in Andenes.

### 2.2 Data processing

Sampled killer whales were identified using nicks, shape and size of the dorsal fin, and scarring and pigmentation patterns of the saddle patch (Bigg, 1982). Individuals were matched to an existing catalogue of 971 killer whales identified between 2007 and 2018 in northern Norway (Jourdain & Karoliussen, 2018). Classification of sex was done as per Bigg (1982). Records of predation on seals collected in 2013–2018 were used to assign sampled killer whales a priori to one of the two diet groups. Individuals with a history of predation on seals were assigned to the group *Seal‐eaters*, while individuals with no such history were classified as *Fish‐eaters*. In addition, individuals were assigned a group reflecting season at sampling. Group *Winter* included individuals sampled at herring wintering grounds in November, group *Spring* contained individuals sampled at lumpfish spawning grounds in April–May and group *Summer* included the whales sampled from June through August in Andfjord (Figure 1).

### 2.3 Stable isotope analysis

Skin samples from killer whales, and muscle from lumpfish, herring, and seal were freeze‐dried and ground individually with an agate mortar and pestle to a fine powder. An aliquot was rinsed three times in a 2:1 chloroform: methanol solution to remove lipids, following the method developed by Folch, Lees, and Stanley (1957) and modified by Elliott, Roth, and Crook (2017). An aliquot from the bulk tissue was not treated with any chloroform: methanol solution. A duplicate analysis was run on nonlipid‐extracted and lipid‐extracted values, in accordance with recommendations (Lesage et al., 2010; Ryan et al., 2012). δ^13^C values were determined from lipid‐extracted samples to control for the low δ^13^C found in the lipid fraction of an organism that can lead to bias (DeNiro & Epstein, 1978; Tarroux et al., 2010; Yurkowski, Hussey, Semeniuk, Ferguson, & Fisk, 2015). δ^15^N values were determined from nonlipid‐extracted samples due to the unpredictable changes in δ^15^N values in fish muscle and cetacean skin following lipid extraction (Lesage et al., 2010; Ryan et al., 2012). The powdered sample (1 mg ± 5%) was weighed into a tin capsule. The δ^15^N and δ^13^C ratios were measured simultaneously using an Elemental Analyzer (EA) IsoLink Isotope Ratio Mass Spectrometer (IRMS) System, consisting of a Flash EA and a DeltaV IRMS (Thermo Scientific, Germany). All analyses were conducted at the Stable Isotope Laboratory at the University of Oslo. The quality of the analysis was assured by the incorporation into each run two internal reference materials, JGLUT (L‐glutamic acid, δ^13^C = −13.43‰, δ^15^N = −4.34‰, Fisher Scientific) and POPPGLY (glycine, δ^13^C = −36.58‰, δ^15^N = 11.25‰, Fisher Scientific). δ^13^C was calibrated to the Vienna Pee Dee Belemnite (VPDB) scale using LSVEC (lithium carbonate, δ^13^C = −46.6‰) and NBS‐ 19 (calcium carbonate, δ^13^C = 1.95‰) (both obtained from the International Atomic Energy Agency, Austria). δ^15^N was calibrated to the AIR scale using USGS40 (L‐glutamic acid, δ^15^N = −4.52‰) and USGS41 (L‐glutamic acid, δ^15^N = 47.57‰) (both obtained from the United States Geological Survey). Analytical precision based on repeated analyses of quality assurance material JALA (alanine, δ^13^C = −20.62‰, δ^15^N = −3.16‰, Fisher Scientific) indicated measurement errors of 0.09 ± 0.01‰ for δ^15^N and 0.06 ± 0.02‰ for δ^13^C.

### 2.4 Statistical analysis

All statistics were performed in R v.3.4.1. for Mac OS X (R Development Core Team, 2017). The α level was set to *p* = .05. To test simultaneously the effect of independent variables sex, diet group (*Seal‐eaters* vs. *Fish‐eaters*), and sampling season group (*Winter*, *Spring,* and *Summer*) on isotopic ratios, a multiple linear regression model was fitted to the data measured for δ^15^N and δ^13^C. Diagnostic plots were used to investigate normality of the residuals, while Levene's test run in the package *car* (Fox & Weisberg, 2011) was used to validate homogeneity of variance in residuals. Significant explanatory variables were identified by conducting a forward model selection in the package *stats*, from the null model to the full global model featuring all variables. A lower Akaike Information Criterion (AIC) was used for model selection, and a higher adjusted R‐squared as an indication of the fit of each model.

A mixture model‐based clustering analysis using the *mclust* package was conducted on the δ^15^N and δ^13^C values to estimate the most likely number of clusters and the probability of individuals belonging to each cluster (Scrucca, Fop, Murphy, & Raftery, 2016). The Bayesian Information Criterion (BIC) was used to select the best model. Individual assignment to clusters was compared to field observations for validation.

The isotopic niche, referring to the isospace delineated by δ^15^N and δ^13^C values, of resulting clusters was estimated using calculated convex hull areas (encompassing all data points) and bivariate ellipses in the package *SIBER* (Jackson, Inger, Parnell, & Bearhop, 2011). The Standard Ellipse Area (SEA) is a measure of the standard deviation for bivariate data. SEA corrected for small sample size (SEA_C_), containing 40% of the data regardless the sample size, enabled robust comparison between clusters. Bayesian Standard Ellipse Areas (SEA_B_) were generated using 10^6^ posterior draws for each cluster and used to statistically compare niche width between clusters (Jackson et al., 2011).

We used stable isotope mixing models (Parnell et al., 2013) in the package *simmr* (Parnell, 2016) to estimate relative contributions of herring, lumpfish, and seal prey to the diet of seal‐eating killer whales at the cluster level. All three prey groups were confirmed to be part of the diet of seal‐eating killer whales from field observations (see Table 1 and Discussion). In a Bayesian framework, and accounting for the putative diet components of a consumer and uncertainties in both food source and consumer isotopic values, models estimate the distributions of possible diets. Killer whale δ^15^N and δ^13^C skin values were input as the consumer data and mean (±*SD*) isotopic values of herring, lumpfish, and seal muscle were used as sources (see Results). To set uncertainty around the single data point available for seal prey, we used published uncertainty data (SD_δ13C_ = 0.2, SD_δ15N_ = 0.3) on isotopic muscle values from stranded harbor seals (*n* = 9) that were collected over a short period of time and from a single region (Hobson, Sease, Merrick, & Piatt, 1997). Concentration dependencies and corrections for trophic discrimination, only optional when running mixing models with *simmr*, were not computed. Due to the large variation in both δ^15^N and δ^13^C values (see Results) among seal‐eating killer whales, mixing models were also run at the individual level using the same segment values.

**TABLE 1:** Summary of the 38 killer whale biopsies sampled in 2017–2018 in northern Norway and used in this study

Whale ID	Sex	Age	Cluster	Diet at sampling	Sampling month	δ^15^N	δ^13^C
(a priori) Fish‐eating individuals							
NKW‐063	Male	Adult	Herring‐eater	Herring	November	11.7	−19.5
NKW‐153	Male	Adult	Herring‐eater	Herring	November	11.7	−19.6
NKW‐165	Male	Adult	Herring‐eater	Herring	November	11.7	−19.4
NKW‐180	Male	Adult	Herring‐eater	Herring	November	11.8	−19.6
NKW‐183	Male	Adult	Herring‐eater	Herring	November	11.9	−19.4
NKW‐421	Male	Adult	Herring‐eater	Herring	November	11.7	−19.0
NKW‐506	Male	Adult	Herring‐eater	Herring	November	11.7	−19.6
NKW‐511	Female	Adult	Herring‐eater	Herring	November	11.4	−19.7
NKW‐862	Male	Adult	Herring‐eater	Herring	November	11.9	−18.9
NKW‐867	Male	Adult	Herring‐eater	Herring	November	11.5	−19.2
NKW‐918	Male	Adult	Herring‐eater	Herring	November	11.5	−19.4
NKW‐919	Male	Adult	Herring‐eater	Herring	November	11.8	−19.8
NKW‐920	Male	Adult	Herring‐eater	Herring	November	11.6	−19.6
NKW‐922	Male	Adult	Herring‐eater	Herring	November	11.7	−19.1
NKW‐923	Male	Adult	Herring‐eater	Herring	November	11.6	−18.9
NKW‐924	Male	Adult	Seal‐eater	Herring	November	12.6	−18.3
NKW‐937	Male	Adult	Herring‐eater	Herring	November	11.9	−19.5
UNK 1	*N*/A	Subadult	Herring‐eater	Herring	November	11.4	−19.2
NKW‐004	Male	Adult	Lumpfish‐eater	Lumpfish	April	11.8	−18.1
NKW‐079	Male	Adult	Lumpfish‐eater	Lumpfish	April	11.5	−17.7
NKW‐348	Male	Adult	Herring‐eater	Lumpfish	April	11.5	−19.2
NKW‐537	Female	Adult	Lumpfish‐eater	Lumpfish	April	11.5	−18.4
NKW‐572	Male	Adult	Lumpfish‐eater	Lumpfish	April	11.5	−18.3
NKW‐714	Male	Subadult	Lumpfish‐eater	Lumpfish	April	11.9	−18.3
NKW‐908	N/A	N/A	Lumpfish‐eater	Lumpfish	April	11.8	−18.2
NKW‐978	N/A	N/A	Lumpfish‐eater	Lumpfish	April	11.3	−18.3
Y137	N/A	Subadult	Lumpfish‐eater	Lumpfish	April	11.5	−18.1
NKW‐598	Male	Adult	Lumpfish‐eater	N/A	July	11.3	−18.6
(a priori) Seal‐eating individuals							
NKW‐785	Female	Adult	Herring‐eater	Herring	November	11.5	−19.2
K1	Female	Adult	Seal‐eater	Lumpfish	May	12.6	−18.5
KI01	Male	Adult	Seal‐eater	N/A	November	12.4	−19.4
KI03	Female	Adult	Seal‐eater	N/A	November	12.3	−20.0
KI05	Male	Adult	Seal‐eater	N/A	November	12.8	−19.2
KI06	Female	Adult	Seal‐eater	Pinnipeds	August	13.2	−18.9
KI07	Male	Adult	Seal‐eater	Pinnipeds	June	12.3	−18.0
NKW‐096	Female	Adult	Seal‐eater	Pinnipeds	August	12.5	−19.1
NKW‐823	N/A	N/A	Seal‐eater	Pinnipeds	August	12.9	−19.4
NKW‐921	Male	Adult	Seal‐eater	Pinnipeds	August	12.5	−18.5

Note

Individuals were listed a priori as fish or seal‐eaters from field observations. Unique identification (ID) codes are given (Jourdain & Karoliussen, 2018), as well as sex and age for each individual when known. Clustering of individuals (*Cluster 1*: seal‐eaters, *Cluster 2*: herring‐eaters, or *Cluster 3*: lumpfish‐eaters), as determined from the maximum likelihood Gaussian mixture model, is indicated. Individuals sampled in November belong to sampling group *Winter*, individuals sampled in April–May belong to sampling group *Spring* and individuals sampled from June through August belong to sampling group *Summer*. Isotopic values refer to lipid‐extracted δ^13^C and nonlipid‐extracted δ^15^N.

Expected δ^15^N and δ^13^C values were also calculated for herring, lumpfish, and seal‐eaters if they were prey specialists using the diet‐to‐tissue skin discrimination factors 1.57 ± 0.52‰ and 1.01 ± 0.37‰ for nitrogen and carbon, respectively, as estimated for bottlenose dolphins by Giménez et al. (2016). The following equations were used, as per Herman et al. (2005):

$$\delta_{N-\text{expected}}=\sum_{i=1}^{n}\left(\text{Diet}\;i\;*\;\delta 15N\;i\right)+1.57$$

$$\delta_{C-\text{expected}}=\sum_{i=1}^{n}\left(\text{Diet}\;i\;*\;\delta 13C\;i\right)+1.01$$

where *n* is the number of different prey species consumed, Diet *i* is the proportion of each prey species consumed, and δ^15^N *i* and δ^13^C *i* are the measured isotopic ratio values of the *i*th prey species being herring, lumpfish, or seal.

## 3 RESULTS

Thirty‐eight individual killer whales were biopsy sampled from 16 encounter days in 2017–2018 in northern Norway (Figure 1, Table 1). Due to dynamic social associations observed throughout the years and challenges to accurately identify stable units, individuals were considered independently except for one case discussed below (see Discussion). For the period 2013–2018, the sampled individuals were encountered throughout the year on one to 23 observation days (mean = 7.8; *SD* = 5.3). Resulting predatory observations confirmed that some of the individuals sampled at matching times and locations adopted different diets (see Jourdain, Karoliussen, et al., 2019; Jourdain et al., 2017; Table 1).

### 3.1 Isotopic values and clustering

Killer whale skin δ^15^N values ranged between 11.3‰ and 13.2‰ (mean ± *SD*: 11.9 ± 0.5‰, *n* = 38) and δ^13^C values ranged between −20.0‰ and − 17.7‰ (−19.0 ± 0.6‰, *n* = 38), resulting in δ^15^N and δ^13^C spanning a range of 1.9‰ and 2.3‰, respectively (Table 1). Herring muscle δ^15^N values ranged between 9.6‰ and 10.4‰ (10.0 ± 0.3‰, *n* = 4) and δ^13^C values ranged between −21.6‰ and −20.6‰ (−21.0 ± 0.5‰, *n* = 4). Lumpfish muscle δ^15^N values ranged between 11.0‰ and 12.3‰ (11.5 ± 0.5‰, *n* = 5) and δ^13^C values ranged between −20.6‰ and −20.3‰ (−20.4 ± 0.1‰, *n* = 5). The one seal muscle sample had a δ^15^N value of 13.9‰ and a δ^13^C value of −19.9‰.

**FIGURE 2:**
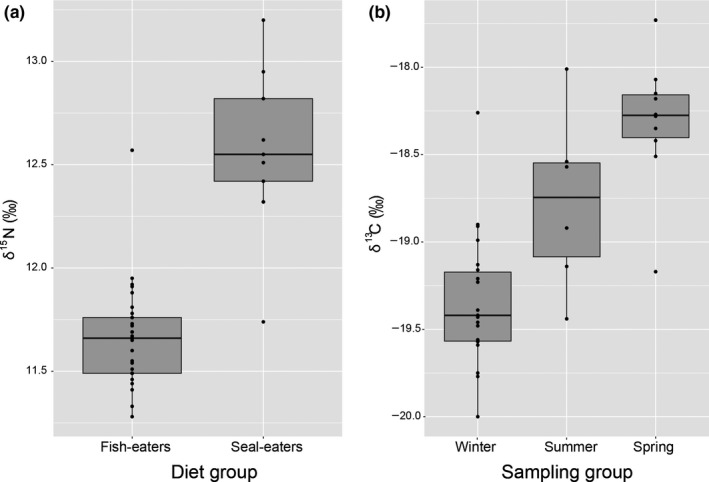
(a) Boxplot of the δ^15^N values (in ‰) measured in skin samples of killer whales known as *Fish‐eaters* (i.e., no history of predation on seals, *n* = 28) and *Seal‐eaters* (i.e., history of predation on seal prey, *n* = 10) from field observations; (b) Boxplot of the δ^13^C values (in ‰) for each of the three sampling groups *Winter*, *Spring,* and *Summer*. For both plots, box represents second and third quartiles, horizontal line corresponds to the median, and whiskers represent the first and fourth quartiles. Data points are represented as dots, and dots outside the box and whiskers are outliers

Multiple linear regression fitted to δ^15^N values, and testing sex, sampling season, and a priori diet groups showed diet group as the only significant explanatory variable (adjusted *R* ^2^ = 0.57, *F*_5,32_ = 10.96, *p* < .001). Whales previously recorded preying on seals had higher δ^15^N values than whales never recorded feeding on seal prey (*Seal‐eaters* vs. *Fish‐eaters*: coefficient estimate = 0.86, *t* = 5.20, and *p* < .001; Figure 2a). The multiple linear regression fitted to δ^13^C showed sampling season as the only significant explanatory variable (adjusted *R* ^2^ = 0.57, *F*_5,32_ = 11, *p* < .001; Figure 2b). Whales sampled at lumpfish spawning grounds in April and May had the highest δ^13^C values (coefficient estimate = −18.31, *t* = −147, *p* < .001), followed by the whales sampled in the summer (*Spring* vs.* Summer*: coefficient estimate = −0.46, *t* = −2.36, *p* = .30). The whales sampled at herring wintering grounds in November showed the lowest δ^13^C values (*Spring* vs. *Winter*: coefficient estimate = −1.02, *t* = −6.86, *p* < .001).

**FIGURE 3:**
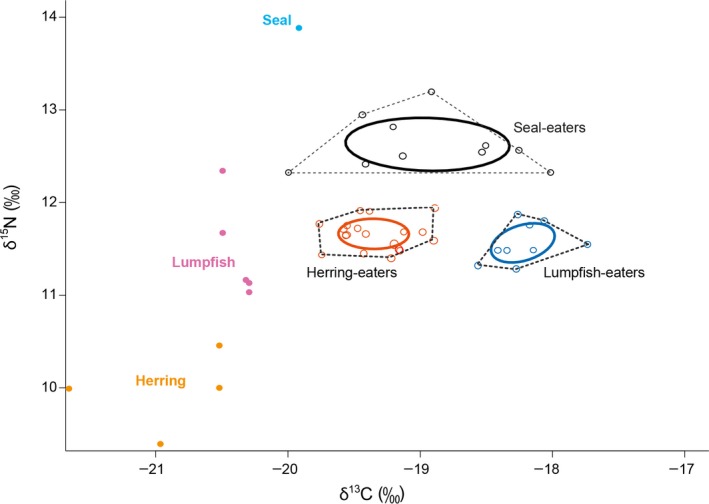
Isospace of δ^15^N and δ^13^C values and niches as measured from killer whale (*n* = 38) skin samples. Seal‐eaters (*n* = 10) are represented as black unfilled circles, herring‐eaters (*n* = 19) as red, and lumpfish‐eaters (*n* = 9) as dark blue. Solid lines represent the standard ellipses corrected for sample size (SEA_C_) and encompassing 40% of the data, while dashed lines represent the convex hull area including the entire dataset for each cluster. Note the absence of overlap between clusters. Filled circles indicate δ^15^N and δ^13^C values from muscle samples of prey (*n* = 4 herring; *n* = 5 lumpfish; *n* = 1 harbor seal)

Based solely on the δ^15^N and δ^13^C values in killer whale skin, the maximum likelihood Gaussian mixture model showed the most likely number of clusters to be three. *Cluster 1* was distinguished by higher δ^15^N values (mean ± *SD*: 12.6 ± 0.3‰, range = 12.3–13.2‰, *n* = 10) than both *Cluster 2* (mean ± *SD*: 11.7 ± 0.2‰, range = 11.4–11.9‰, *n* = 19) and *Cluster 3* (mean ± *SD*: 11.6 ± 0.2‰, range = 11.3–11.9, *n* = 9). *Cluster 1* showed a wide variation in δ^13^C with values intermediate to *Cluster 2* and *Cluster 3* (Table 1, Figure 3). Except for the three individuals NKW‐348, NKW‐785, and NKW‐924 assigned to unexpected clusters (see Table 1), assignment of individuals to clusters coincided largely with predatory field observations (Jourdain, Karoliussen, et al., 2019; Jourdain & Vongraven, 2017; Jourdain et al., 2017; Table 1; Figure 3). *Clusters 1:* seal‐eaters, *Cluster 2:* herring‐eaters, and *Cluster 3:* lumpfish‐eaters were therefore used for the isotopic analyses hereafter.

Average δ^15^N values measured in individuals from *Cluster 1:* seal‐eaters were approximately 1‰ higher than in fish‐eating individuals from *Cluster 2:* herring‐eaters and *Cluster 3:* lumpfish‐eaters combined (mean ± *SD*: 11.6 ± 0.2‰, range = 11.3–11.9, *n* = 28).

### 3.2 Isotopic niche and diet composition

Niche width based on isotopic signatures for herring and lumpfish‐eating killer whales appeared narrow and not significantly different as supported by a similar SEA_B_ (*p* = .14; Figure 3). SEA_B_ for seal‐eaters indicated a wider niche than both clusters (*p* < .001) as supported by a wide convex hull (Figure 3). Total absence of SEA_C_ overlap among groups suggested that the three dietary clusters occupy distinct niches (Figure 3).

**FIGURE 4:**
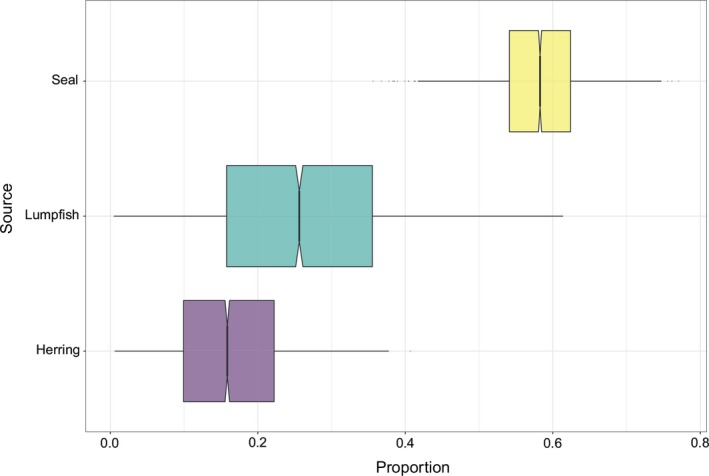
Outputs from Bayesian isotopic mixing models showing proportional estimated contributions (mean, 25% and 75% percentiles) of herring, lumpfish, and harbor seal to the diet of seal‐eating killer whales (*Cluster 1*, *n* = 10) biopsy sampled in northern Norway

**FIGURE 5:**
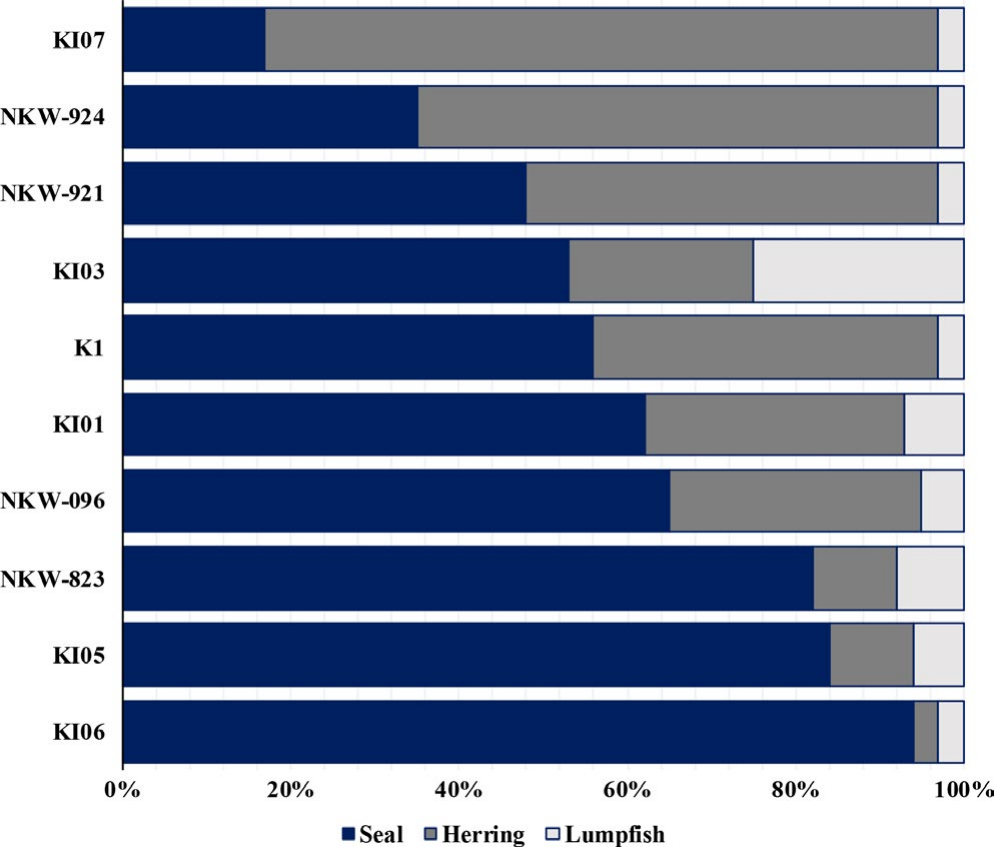
Individual variation in the mean proportional contribution of harbor seal, herring, and lumpfish to the diet of seal‐eating killer whales (*Cluster 1*, *n* = 10) biopsy sampled in northern Norway, as estimated from Bayesian isotopic mixing models

Bayesian stable isotope mixing models estimated mean relative contributions of the three prey groups to the diet of seal‐eating killer whales to be: herring (mean ± *SD*) = 0.16 ± 0.08, lumpfish = 0.26 ± 0.12, and harbor seal = 0.58 ± 0.06 (Figure 4). Mixing models run separately for each seal‐eating individual further suggested a large variation in proportional contribution of harbor seal compared to herring and lumpfish. For seven of the 10 seal‐eating killer whales, harbor seal was the highest dietary contributor although fish prey also appeared as a significant food source (Figure 5). Mean contribution of harbor seal ranged from 17% (2.5%–97.5% quantiles: 2%–36%, whale KI07) to 93% (85%–98%, whale KI06, Figure 5). Expected values for potential prey specialists feeding on herring, lumpfish, and seals are shown in Table 2.

**TABLE 2:** Calculated expected δ^15^N and δ^13^C skin values for killer whales feeding exclusively on herring, lumpfish, and seal prey as compared to mean true (measured) values for each dietary cluster

	Expected δ^15^N; δ^13^C (in ‰)	True values δ^15^N; δ^13^C (in ‰)
*Cluster 1:* seal‐eaters	15.5; −18.9	12.6; −18.9
*Cluster 2:* herring‐eaters	11.6; −20.0	11.7; −19.3
*Cluster 3:* lumpfish‐eaters	13.0; −19.4	11.6; −18.2

## 4 DISCUSSION

Recent killer whale studies in Norway suggested multiple prey resources (Cosentino, 2015; Jourdain, Karoliussen, et al., 2019; Jourdain et al., 2017; Nøttestad et al., 2014; Vester & Hammerschmidt, 2013; Vongraven & Bisther, 2014) as opposed to initial observations of killer whales being herring specialists in this region (Christensen, 1982; Similä et al., 1996). Our results further support a generalist population characterized by interindividual dietary variations. Low variation in δ^15^N and δ^13^C skin values for killer whales sampled at herring and lumpfish grounds supported previous field observations of seasonal specialization on abundant fish prey (Jourdain, Karoliussen, et al., 2019; Similä et al., 1996; Similä & Ugarte, 1993). Higher δ^15^N skin values measured throughout the year for individuals repeatedly observed feeding on seals in 1988–2016 (Jourdain et al., 2017; Vongraven & Bisther, 2014) supported persistent feeding at higher trophic level and marked preference for pinniped prey for these whales, though a wide isotopic niche indicated a diversified diet.

Herring and lumpfish‐eating killer whales had similar δ^15^N values, indicative of feeding at comparable trophic levels. Low variation in δ^15^N and δ^13^C values in herring‐eaters (*Cluster 2*) resulted in the narrowest niche of the three clusters, implying consistency in diet among individuals and apparent prey specialization in the winter months of high herring abundance. This is consistent with Foote, Vester, Vikingsson, and Newton (2012) who found only little variation in isotopic values (mean ± *SD*: 11.8 ± 0.5, range = 10.9–12.9, *n* = 20) in killer whales sampled at herring wintering grounds in northern Norway in 2005 and 2007. Lumpfish‐eating killer whales (*Cluster 3)*, sampled in the first half of April in Andfjord, were shown to seasonally feed on lumpfish, based on predatory observations and molecular identification of target prey (Jourdain, Karoliussen, et al., 2019). These whales showed a slightly wider isotopic niche, possibly indicative of a gradual seasonal switch in diet, from herring to lumpfish. As the wintering herring initiates its progressive migration southwards to spawning grounds at the end of January in the study area (Røttingen, 1990), the lumpfish migration from offshore feeding areas to inland spawning grounds starts in February (Eriksen, Durif, & Prozorkevich, 2014). Such overlap in time and space between the two fish species could promote temporary inclusion of both fish prey in killer whales’ diet. Indeed, sampled lumpfish‐eating killer whales were confirmed to be feeding on herring from winter encounters between 2015 and 2018 (Jourdain, Karoliussen, et al., 2019). In any case, seasonal specialization on lumpfish is likely reflecting a seasonal local peak in prey abundance rather than true dietary preference.

A few individuals sampled at herring grounds (i.e., whales KI01, KI03, KI05, and NKW‐924) and lumpfish grounds (i.e., whale K1) showed higher δ^15^N skin values, indicative of feeding on higher trophic level prey. These whales were assigned to *Cluster 1*: seal‐eaters for which distinctive enrichment in ^15^N correlated with apparent inclusion of seals in their diet. This was confirmed from field observations for nine of the 10 whales in this cluster (see Jourdain et al., 2017). Seal‐eaters showed δ^15^N skin values in average 1.0‰, and up to 1.9‰, higher compared to fish‐eaters, regardless of sampling season. These values are consistent with approximately one trophic level, assuming an estimated discrimination factor of ~1.57‰ for nitrogen for bottlenose dolphin (Giménez et al., 2016). This falls within or exceeds the range of values discriminative of fish versus marine mammal‐eating killer whales in Iceland (ΔN = 1.32‰ in Samarra et al., 2017), British Columbia/Washington State (ΔN = 0.7‰, in Herman et al., 2005), Eastern Aleutian Islands (ΔN = 1.2‰ in Herman et al., 2005), and Antarctica (ΔN = 0.9‰ in Durban et al., 2017). Disparate isotopic profiles among individuals sampled at matching times and locations in our study indicate co‐occurrence of individuals that include, and others that do not include, pinnipeds to their diet. Elevated δ^15^N values found in three of the 20 killer whales sampled at herring wintering grounds by Foote et al. (2012) also suggested such dietary structuring.

Combined with individual predation records collected over years and up to several decades (see Jourdain et al., 2017; Vongraven & Bisther, 2014), our results confirm persistent dietary preference rather than opportunistic feeding on pinnipeds for these whales. Consistently elevated δ^15^N values for all ten seal‐eaters (*Cluster 1*) sampled in all seasons further indicated that predation on seals (or other high trophic level prey) occurred throughout the year, even at times of high abundance of fish prey. This was supported by strikingly similar skin δ^15^N values measured for individuals KI01, KI03, and KI07. All three individuals were shown to constitute a stable long‐lasting social group that hunts and feeds cooperatively (Jourdain et al., 2017), and therefore, comparable diet and isotopic profiles are expected for these whales. Although sampled in different seasons for this study (see Table 1), year‐round homogeneous δ^15^N values support consistent feeding habits for this group regardless of the time of the year.

A wider isotopic niche found for seal‐eating killer whales compared to fish‐eaters implies a more diversified diet. This was supported by the following observations of seal‐eating individuals also feeding on fish prey. Adult males NKW‐924 and KI01 were photographed while scavenging around herring‐purse seiners in November 2017 and 2018 (EJ, unpublished data), respectively, confirming effective inclusion of herring to their diets. Whales KI03, KI05, and KI06 were also observed carousel feeding on herring off Ona, Møre coast, Norway in 1991, immediately prior to travelling near‐shore to chase harbor seals (Vongraven & Bisther, 2014). Female K1 was observed feeding on lumpfish at time of sampling in May 2018 before resuming nearshore foraging as previously described for seal‐eating groups (Jourdain et al., 2017). Expected δ^15^N values calculated for seal‐eaters if they were prey specialists were in further support of a mixed diet (Table 2).

When computing herring, lumpfish, and harbor seal as putative prey of seal‐eating killer whales (*Cluster 1*), isotope mixing models suggested that seal prey made up 46 to 68% of these whales’ diet (2.5 and 97.5% quartiles at the cluster level), therefore suggesting fish prey as an equal or secondary food source (Figure 4). However, these results should only be considered preliminary and interpreted with caution due to small sample size for both killer whale and prey values, and due to the limitations and assumptions involved in the use of these models. Mixing models are very sensitive to the assumption that all potential prey sources are computed (Bond & Diamond, 2011; Parnell et al., 2013; Phillips et al., 2014), which can be confirmed if isotopic values of the consumer fall within the mixing polygon of the connecting food sources once corrected for trophic fractionation (Phillips & Koch, 2002). This was not the case here (see Figure 3), implying missing prey sources. Also, if there was large temporal and/or individual variation in diet composition; that is, not all seal‐eating individuals in *Cluster 1* feeding on all three prey types, and not at time of sampling, computing herring, lumpfish, and seal prey could be unsuitable (Phillips et al., 2014; Phillips & Koch, 2002). Another caveat in our models resides in the use of variation values from harbor seals that were not collected in our study area. Further efforts in monitoring food sources to better obtain a priori knowledge on individuals’ diet, and collecting prey samples, are warranted. More complete datasets and inclusion of appropriate trophic discrimination factors would allow for more accurate diet reconstruction (Phillips et al., 2014) of seal‐eating killer whales in Norway and should be a priority for future research.

Nevertheless, the large variation observed in estimated dietary contributions among seal‐eating killer whales is consistent with the wide isotopic niche that characterizes this cluster (Figures 3, 4, 5). Importantly, there might be a gradient of dietary patterns among Norwegian killer whales ranging from fish specialists (*Clusters 2* and *3*: herring and lumpfish‐eaters) on one end to individuals that feed most extensively on high trophic level prey (*Cluster 1:* seal‐eaters) on the other end. Contrasting field observations and isotopic values for whale NKW‐785 support this theory. This female was observed feeding on pinnipeds on one encounter day in June 2017 (EJ unpublished data) and feeding on herring at time of sampling in November of the same year. Owing to low δ^15^N skin values, this whale was assigned to the cluster herring‐eaters by the Gaussian mixture model, bringing evidence that not all killer whales known as seal‐eaters from field observations forage on pinnipeds throughout the year. Instead, some individuals may seasonally switch between specialized feeding behaviors or may only opportunistically feed on seals.

Variations in δ^13^C among the three sampling groups support variable foraging areas in relation to seasonal prey movement and/or foraging on prey that utilize different habitats. The lowest δ^13^C values measured for herring‐eaters (*Cluster 2*) sampled in November at herring wintering grounds coincide with killer whales just returning from their summer offshore distribution (Nøttestad et al., 2014, 2015), following the migration of the herring to the coastal wintering grounds in early fall. Lower δ^13^C values could also be a result of specializing on herring, which is a pelagic fish spending most of its life offshore (Dragesund, Hamre, & Ulltang, 1980). The highest δ^13^C values measured in killer whales sampled at lumpfish spawning grounds in spring (*Cluster 3*) were indicative of a coastal habitat for these whales. This is consistent with a winter spent in fjords foraging on wintering herring but could also be a result of temporarily specializing on the lumpfish which is a semipelagic fish (Davenport, 1985). Intermediate δ^13^C values for whales sampled from June through August could indicate intergroup variations in foraging areas due to a mosaic of prey resources (Jourdain et al., 2017; Nøttestad et al., 2014; Similä et al., 1996) relied upon at this time of the year.

Expected values of δ^15^N and δ^13^C in skin of killer whales that would exclusively feed on seal prey indicate that pinniped‐eating individuals sampled in this study are not prey specialists. Vongraven and Bisther (2014) suggested that the near total collapse of the NSS herring in 1970 caused by overfishing, could have forced a herring‐dependent population of killer whales to switch to other prey types including pinnipeds. Phenotypic plasticity and the ability to learn and culturally transmit new hunting techniques may have facilitated such a switch (Riesch et al. 2012; Samarra & Miller, 2015). Further resource specialization may increase foraging efficiency if experienced foragers benefit from enhanced searching and handling abilities of better selected cost‐effective prey (see Bolnick et al., 2003). Under consistent environmental conditions, and if prey specialists indeed experienced a greater fitness than generalists, the level of prey specialization could increase over time regardless of target prey, as shown in other species (Annett & Pierotti, 1999; Golet, Kuletz, Roby, & Irons, 2000).

Despite a small sample size, our study captured a diversity of dietary patterns largely consistent with field observations. Results highlight dietary structuring and differences in prey specialization within this killer whale population which could reflect either seasonal localized food abundance, individuals’ true dietary preferences, or both. Resampling of individuals over time and throughout the year would assist in assessing intraindividual dietary variations. While links between diet, genetics and social structure remain to be investigated in this region, our observations confirm that at least seasonal range overlap occurs among killer whale groups adopting distinct diets.

## CONFLICT OF INTERESTS

Authors have no competing interests to declare.

## AUTHOR CONTRIBUTIONS

E.J., C.A., R.K., and D.V. conceptualized the study. E.J., C.A., and R.K. collected samples. E.J., C.A., A.R., and K.B. conceptualized the analyses. C.A. conducted laboratory work. E.J. and C.A. analyzed data. E.J., C.A., and D.V. wrote the paper. All authors approved the final manuscript.

## Data Availability

All isotopic values used in this study are available from Figshare Digital Repository: https://doi.org/10.6084/m9.figshare.11901933.v1
